# Level of Sulfite Oxidase Activity Affects Sulfur and Carbon Metabolism in *Arabidopsis*

**DOI:** 10.3389/fpls.2021.690830

**Published:** 2021-06-24

**Authors:** Dinara Oshanova, Assylay Kurmanbayeva, Aizat Bekturova, Aigerim Soltabayeva, Zhadyrassyn Nurbekova, Dominic Standing, Arvind Kumar Dubey, Moshe Sagi

**Affiliations:** ^1^The Albert Katz International School for Desert Studies, The Jacob Blaustein Institutes for Desert Research, Ben-Gurion University of the Negev, Beersheba, Israel; ^2^The Albert Katz Department of Dryland Biotechnologies, French Associates Institute for Agriculture and Biotechnology of Dryland, The Jacob Blaustein Institutes for Desert Research, Ben-Gurion University of the Negev, Beersheba, Israel; ^3^Jacob Blaustein Center for Scientific Cooperation, The Jacob Blaustein Institutes for Desert Research, Ben-Gurion University of the Negev, Beersheba, Israel

**Keywords:** sulfite network, sulfite oxidase, sulfite reductase, carbon, sulfur metabolism

## Abstract

Molybdenum cofactor containing sulfite oxidase (SO) enzyme is an important player in protecting plants against exogenous toxic sulfite. It was also demonstrated that SO activity is essential to cope with rising dark-induced endogenous sulfite levels and maintain optimal carbon and sulfur metabolism in tomato plants exposed to extended dark stress. The response of SO and sulfite reductase to direct exposure of low and high levels of sulfate and carbon was rarely shown. By employing *Arabidopsis* wild-type, sulfite reductase, and SO-modulated plants supplied with excess or limited carbon or sulfur supply, the current study demonstrates the important role of SO in carbon and sulfur metabolism. Application of low and excess sucrose, or sulfate levels, led to lower biomass accumulation rates, followed by enhanced sulfite accumulation in SO impaired mutant compared with wild-type. SO-impairment resulted in the channeling of sulfite to the sulfate reduction pathway, resulting in an overflow of organic S accumulation. In addition, sulfite enhancement was followed by oxidative stress contributing as well to the lower biomass accumulation in SO-modulated plants. These results indicate that the role of SO is not limited to protection against elevated sulfite toxicity but to maintaining optimal carbon and sulfur metabolism in *Arabidopsis* plants.

## Introduction

Molybdenum-containing sulfite oxidase is an enzyme primarily responsible for catalyzing sulfite oxidation to sulfate. Genetic deficiency of human sulfite oxidase leads to severe neurological abnormalities that often result in death in infancy (Johnson and Wadman, 1995; Garrett et al., 1998). Among eukaryotes, plant sulfite oxidase (SO; EC 1.8.3.1) is the smallest molybdenum co-factor-containing enzyme known so far. In *Arabidopsis*, AtSO is localized to the peroxisomes and catalyzes the oxidation of sulfite, the intermediate product of sulfate assimilation (Eilers et al., 2001). Assimilatory reduction of soil-available sulfate is the main pathway of sulfite acquisition in plants, where sulfate is first activated by ATP sulfurylase (ATPS, EC 2.7.7.4), resulting in adenosine-5′-phosphosulfate (APS) that is reduced to sulfite by the plastid localized APS reductase enzymes [APR; EC 1.8.4.9 (Kopriva and Koprivova, 2004; Takahashi et al., 2011)]. The sulfite is then reduced by sulfite reductase (SiR; EC 1.8.7.1) to produce the reduced sulfide form for incorporation into sulfur-containing amino acids (Nakayama et al., 2000; Khan et al., 2010).

In addition to SO and SiR, plants can perform alternative sulfite conversion. The chloroplast localized UDP-sulfoquinovose synthase1 (SQD1; EC 3.13.1.1) utilizes sulfite for the biosynthesis of sulfoquinovosyl diacylglycerols (SQDGs; sulfolipids) required for the proper function of the photosynthetic membranes (Sanda et al., 2001). Alternatively, sulfite can be detoxified through a multi-gene family of 21 sulfur transferases [STs; EC 2.8.1.2 (Papenbrock et al., 2011; Moseler et al., 2019)], which participate in sulfite detoxification, generating the less toxic thiosulfate; or catalyze the back reaction and produce sulfite by sulfur transfer to cyanide, resulting in thiocyanate. Produced thiosulfate might be further metabolized into hydrogen sulfide *via* STs through *in vivo* interactions with thioredoxins [Trx (Henne et al., 2015)] as shown before with the mammalian 3-MP Str (Mikami et al., 2011).

Most studies highlight the role of SiR and SO in exogenously applied sulfite detoxification as the two main sulfite consumers (Brychkova et al., 2007; Lang et al., 2007; Randewig et al., 2012; Yarmolinsky et al., 2013). SiR functions as a “bottleneck” of the sulfate reduction pathway (Khan et al., 2010), while SO functions as the “safety valve” for detoxifying excess amounts of sulfite (Brychkova et al., 2007; Hansch et al., 2007). Both SiR and SO enzymes were shown to play crucial roles in enabling *Arabidopsis* and tomato plants to cope with excess sulfite, exhibiting that mutants of these genes were sensitive to toxic sulfite levels while plants with overexpressed activities of the gene products were more tolerant (Brychkova et al., 2007; Lang et al., 2007; Randewig et al., 2012; Yarmolinsky et al., 2013).

The response of SO to direct exposure to low and high levels of sulfate and carbon was rarely shown. Exposing tomato WT and SO Ri mutant plants to dark-induced carbon starvation, it was shown that SO activity is essential to maintain optimal carbon and sulfur metabolism. SO activity was shown to play an essential role in protecting plants against internal sulfite overflow maintaining the sulfite homeostasis in plants exposed to extended dark stress (Brychkova et al., 2015). However, this was done indirectly under unnatural conditions of extended dark stress (more than 10 days in the dark), causing carbon starvation and sulfate assimilation starvation dependent on light (Schmidt and Trebst, 1969; Kopriva et al., 1999). To further demonstrate the role of SO in carbon and sulfur metabolism under biologically relevant conditions, *Arabidopsis* wild-type (WT), SiR, and SO-modulated plants were exposed for 9 days to carbon and sulfate treatments. Application of starve/excess carbon and sulfate led to lower biomass accumulation in SO compared with WT. The result of accumulated toxic sulfite levels led to the futile channeling of excess sulfite reduction, resulting in over-accumulation of thiols and energy waste. The results indicate that SO activity is necessary to handle increased endogenous sulfite levels and plays an essential role in optimizing sulfur and carbon metabolism.

## Results

### Response of SiR- and SO-Modulated Plants to Excess and Low Sulfate Application

**Figure 1:**
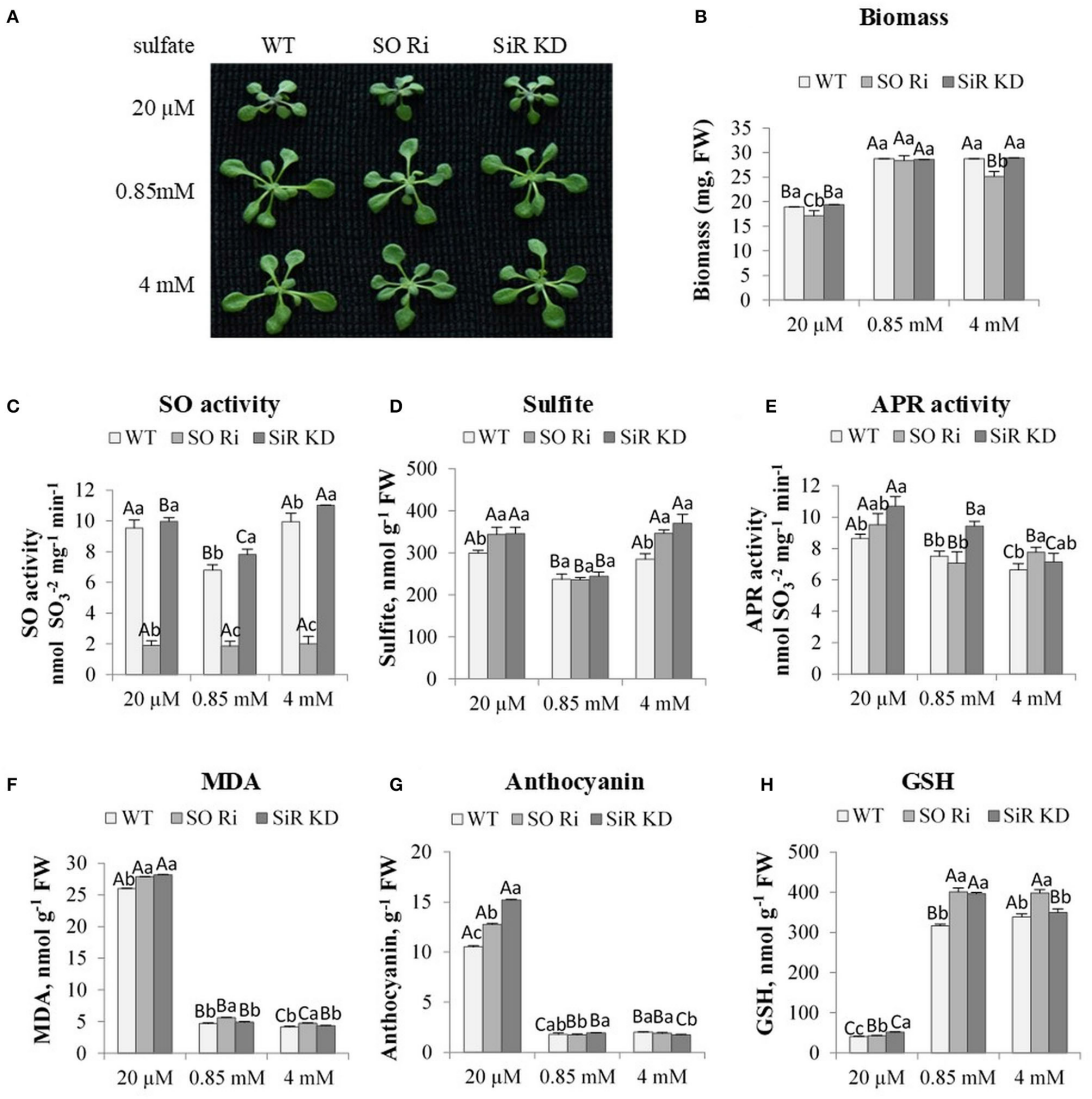
The effect of starvation (20 μM), normal (0.85 mM) and excess (4 mM) sulfate treatment on biomass accumulation, SO activity, S containing metabolites and oxidative stress indicators levels in wild-type (WT), sulfite reductase (SiR), and sulfite oxidase (SO) impaired plants. **(A)** Plants appearance. Plants were photographed on the ninth day after transfer to 0.5 MS plates containing the supplemented sulfate as the only S source. **(B)** Total biomass accumulation of plants upper part. Values are means (*n* = 20 plants) in one of six independent experiments with similar results. **(C)** SO activity. **(D)** Sulfite level. **(E)** APR activity. **(F)** Malondialdehyde (MDA) (the product marker of lipid peroxidation), **(G)** Anthocyanin, and **(H)** Reduced glutathione (GSH) level. Values for **(C,H)** represents one of three independent experiments with similar results (±SE, *n* = 3). Values for **(D–G)** represent the means of three independent experiments (±SE, *n* = 3). Different lower-case letters indicate differences between genotypes within the same treatment. Different uppercase letters indicate significant differences within the plant genotypes in response to treatment (Tukey–Kramer HSD test; JMP 8.0 software).

To examine the role of SO in sulfur metabolism, WT, SO RNA interference (SO Ri) and SiR knockdown (SiR KD) impaired plants were grown for 9 days in plates containing 0.5% MS supplemented with 20 μM, 0.85 mM, or 4 mM sulfate as the only S source. The limited (20 μM) sulfate treatment led to a lower accumulation rate of biomass in all three genotypes, compared with the plants grown under normal (0.85 mM) sulfate level (Figures 1A,B). These results agree with the notion that sulfur starvation results in reduced growth (Gilbert et al., 1997; Hirai et al., 2003). Among the three genotypes grown on limited sulfate, SO Ri showed significantly lower biomass accumulation than WT and SiR KD. Similarly, the application of excess sulfate resulted in a significant reduction of total biomass in plants with impaired SO activity compared with SiR KD and WT plants (Figure 1B). The lowest biomass accumulation rate was of plants with impaired SO under 20 μM and 4 mM sulfate. In contrast, no differences were noticed in plants grown with 0.85 mM sulfate, indicating that among the three sulfate levels examined, the normal SO level is optimal for controlling plant growth, likely by optimal sulfur metabolism.

In the absence of SO activity, above a certain threshold of sulfite level, the accumulation of sulfite in a short-term exposure (2 h to 3 days) was accompanied by increased leaf damage and even planted mortality (Brychkova et al., 2007; Lang et al., 2007; Randewig et al., 2012). Notably, the SO activity rate was higher in SiR KD, and WT in plants supplied with limited sulfate than control plants was lower only compared with WT and SiR KD fed with excess sulfate (Figure 1C). Enhanced, SO activity followed the sulfite up-regulation in SiR KD mutant and WT plants under sulfate starvation and excess sulfate compared with the normal sulfate condition (Figure 1D). The enhanced sulfite level under low sulfate supply is likely the result of APR enhanced activity in all genotypes compared with plants grown under normal sulfate level (Figure 1E). This is likely the result of oxidative stress shown to induce the enhancement of APR (Bick et al., 2001; Koprivova et al., 2008). The consequences of the oxidative stress are noticed by the high level of the lipid peroxidation product, the malondialdehyde (MDA), as well as the level of the antioxidant's anthocyanins [Figures 1F,G (Gould et al., 2002; Xu et al., 2017)].

**Figure 2:**
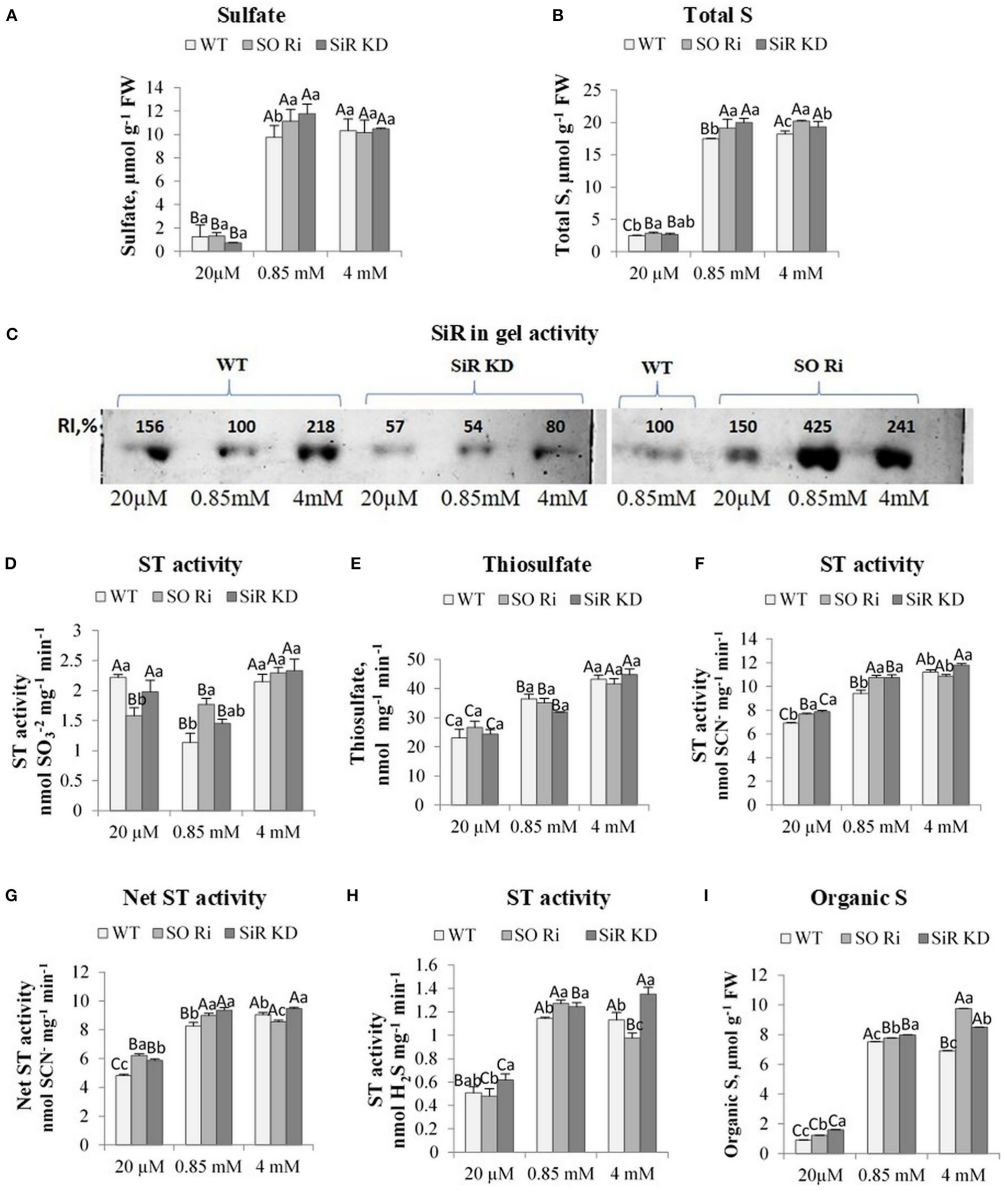
The effect of starvation (20 μM), normal (0.85 mM), and excess (4 mM) sulfate treatment on the levels of sulfate, thiosulfate, total, and organic S, sulfite reductase (SiR), and sulfurtransferases (STs) activities in wild-type (WT) and (SiR) and sulfite oxidase (SO) impaired plants. **(A)** Sulfate and **(B)**. Total sulfur (S) levels. **(C)** SiR in-gel activity after crude protein extracts fractionation by NATIVE-PAGE. Each lane contained 10 μg of proteins. Two separate gels with three treatments including control detected together are presented. The crude proteins extracted from WT leaves in plants treated with 0.85 sulfate was loaded to the two gels, and the intensity of the detected SiR activities was used as the control (100%) for each of the two gels. RI represents relative intensity (%) to the activity of WT treated with 0.85 sulfate. **(D)** STs sulfite consumption activity. **(E)** Thiosulfate level. **(F)** STs sulfite producing activity. **(G)** The net STs activity (calculated as the difference between sulfite-producing and sulfite-consumption activities). **(H)** STs sulfide generation activity. **(I)** Total organic S [calculated as total-S minus (sulfate plus sulfite)]. Values for **(A,B,D–F,H)** represent the means of three independent experiments (±SE, *n* = 3). **(C)** Represents one of three independent experiments with similar results. Different lowercase letters indicate differences between genotypes within the same treatment. Different uppercase letters indicate significant differences within the plant genotypes in response to treatment (Tukey–Kramer HSD test; JMP 8.0 software).

Interestingly, the same level of sulfite was accumulated under the excess amount of sulfate, despite the lower APR activity rate compared with the level in plants grown with the starvation level of sulfate (Figures 1D,E), indicating additional factors may affect sulfite levels in leaves. The reliability of the high sulfite level in SiR KD grown on high sulfate is supported by the enhanced SO activity in this mutant (Figure 1C). Additionally, it is supported as well by the highest sulfite consuming activity rate of the STs that generated thiosulfate, followed by the enhanced sulfide generation activity by STs when employing thiosulfate as the substrate for SiR KD supplemented by the highest sulfate levels (see below in Figures 2D,H). This points to another source of sulfite production other than APR. This might be due to the enhanced degradation as well as oxidation of the high thiol levels accumulated, resulting in enhancement of oxidized glutathione (GSSG) level with enhanced sulfate application (Supplementary Figures 1A,B), as well as the sulfide generated by SiR and/or STs (see below, Figures 2C,H). GSSH, the outcome of sulfide and GSSG interaction, is the substrate for generating sulfite, catalyzed by the mitochondrial sulfur dioxygenase ethylmalonic encephalopathy protein1 [ETHE1 (Krüßel et al., 2014)]. STs could also generate sulfite (see below, Figures 2F,G). In support of this notion, examining ETHE1 gene expression in response to sulfate revealed the increase in all genotypes fed with 20 μM sulfate compared with 0.85 mM and 4 mM of sulfate sources (Supplementary Figure 1C).

Additionally, ETHE1 was significantly higher in SO Ri than the other genotypes in all three sulfate levels applied, whereas SiR KD was higher than WT at the lowest and highest sulfate applied. These results indicate that the source of sulfite under the lowest sulfate level is likely the consequence of both sulfite generating enzymes: APR and ETHE1, in SO Ri and SiR KD under low and excess sulfate (Figure 1E, Supplementary Figure 1C). However, STs cannot be ruled out as one of the players responsible for the accumulated sulfite (see below, Figure 2F).

To prevent sulfite toxicity, SO acts as a safety valve detoxifying excess sulfite, homeostatic in the sulfate reduction pathway (Brychkova et al., 2007; Lang et al., 2007) and the sulfite generated by ETHE1 and STs activities (Papenbrock et al., 2011; Krüßel et al., 2014). Whereas, SiR and SO mutant plants showed significantly higher accumulated sulfite compared with WT in plants treated with the lowest and highest sulfate, the level of sulfate as the result of sulfite oxidation and/or uptake from the growth medium was equal between genotypes within low and excess sulfate treatments (Figure 2A). In contrast, a higher sulfate accumulation rate than WT was noticed in SO Ri and SiR KD mutant plants grown under normal sulfate levels. Sulfate enhancement in SO Ri mutant is mostly the result of enhanced sulfate uptake (Brychkova et al., 2007), and in SiR, KD is likely the consequence of both: sulfite oxidation by SO and sulfate uptake. The similar sulfate levels accumulated in control and excess sulfate grown plants may indicate a feedback regulation of sulfate uptake by enhancing thiols under the excess sulfate condition (Figures 1H, 2A, Supplementary Figure 1A). Indeed, GSH level was shown to affect sulfate transporters expression (Lappartient et al., 1999; Takahashi et al., 2000). Interestingly, higher total sulfur concentrations were detected in the mutants compared with WT plants in all three levels of sulfate growth conditions, indicating that SiR and SO impairment resulted in enhanced sulfate uptake compared with WT plants (Figure 2B).

### SiR and STs in Sulfate Treated WT, SO Ri, and SiR KD Plants

The accumulated sulfite in plants can be oxidized to sulfate and the less toxic thiosulfate or reduced to sulfide for further Cys biosynthesis through the reduction pathway (Leustek and Saito, 1999; Nakayama et al., 2000; Tsakraklides et al., 2002), where SiR is recognized to be a bottleneck in the reductive sulfate pathway (Khan et al., 2010). Employing in-gel SiR activity demonstrated that the suppression of SO activity relative to the WT grown under normal sulfate conditions was followed by increased SiR activity at all three levels of supplied sulfate (Figure 2C). SiR in-gel activity rate in SO impaired plants was 1.5-, 4.2-, and 2.4-fold higher under starvation, normal, and excess sulfate supply, respectively, as compared with WT under normal sulfate supply. GSH levels followed the higher intensity of SiR in SO Ri mutant plants in all supplied sulfate levels, exhibiting a significantly higher level than WT leaves (see above, Figure 1H). These results show the capacity of SO-impaired plants to protect themselves against toxic sulfite through sulfite reduction by SiR activity, resulting in enhanced GSH. However, the active SiR in SO Ri plants was not enough to prevent a certain level of sulfite accumulation. Notably, the detoxification of sulfite *via* SiR in the absence of active SO seems to be metabolically costly and would result in a futile pathway generating excess unnecessary thiols from which sulfite might again be released as a result of sulfur-containing metabolite turnover (Tsakraklides et al., 2002; Takahashi et al., 2011).

Interestingly total thiol in SiR KD was accumulated at the same level of SO mutant plants under the normal and excess amount of sulfate (Supplementary Figure 1A). The accumulated thiols in SiR impaired plants indicate possible sulfite utilization by bypassing the sulfate reduction pathway through a pathway recruiting alternative sulfite consumers such as the ST to generate sulfide when the expression of SiR is fully or partially blocked.

The activity of sulfur transferases in SO Ri and SiR KD compared with WT was studied to examine the capacity of the STs to participate in sulfite detoxification and homeostasis. The nuclear genome of *Arabidopsis* was reported to encode 20 STs sequences (Bauer and Papenbrock, 2002; Papenbrock et al., 2011), and recently an additional protein containing an Rhd domain was added as AtST19 (Moseler et al., 2019). Many different STs have been shown to catalyze reactions that result in sulfite generation or consumption and sulfide production, depending on the substrate used (Papenbrock et al., 2011). The detection of STs activities represents the sum of the activities of the STs group members. The sulfite-consuming activity was detected as sulfite disappearance in the presence of thiocyanate (SCN^−^), abolishing the interference of sulfite consumption activity by sulfite oxidase by inhibiting SO with sodium orthovanadate as described before (Kaufholdt et al., 2015). Measurement of STs activities in sulfite consumption showed upregulation in WT and SiR KD grown under sulfate starvation and all three genotypes grown with excess sulfate compared with the control condition (Figure 2D). Notably, the level of thiosulfate, the product of sulfite consumption by STs, was increased in the two mutants with the sulfate-enhanced application (Figure 2E). However, the thiosulfate was lower under the lowest amount of sulfate in all genotypes, likely the result of S starvation.

Interestingly sulfite generation activity rate by STs utilizing thiosulfate as the substrate was induced by increasing sulfate application in all genotypes except SO Ri under excess sulfate (Figure 2F). Sulfite generation was higher than sulfite consumption activity, being upregulated in both mutants fed with low and normal sulfate and in SiR KD under excess sulfate compared with corresponding WTs (Figure 2F). The net STs activity is defined as the difference between sulfite-producing activity and sulfite-consumption activity, followed by sulfite generation activity (Figure 2G). Notably, the sulfide production activity by STs employing thiosulfate as the substrate was enhanced with sulfate level applied and was higher in SiR KD mutant than WT in plants with normal and excess sulfate (Figure 2H). However, the highest sulfide generation activity rate of SO Ri was evident under normal growth conditions (Figure 2H). These results indicate that STs can participate in sulfite detoxification and its homeostasis by the generation of sulfide to compensate for SiR and SO impairment and protect the cells from sulfite toxicity. Notably, similar to thiol enhancement (Supplementary Figure 1A), both mutants exhibited increased organic-S with sulfate enhancement, the highest in SO Ri treated with 4 mM sulfate, whereas both SiR KD and SO Ri contained higher organic-S than WT (Figure 2I). These can be explained by increasing sulfide generation activity by SiR and STs in SO Ri and *via* STs enhancement in SiR KD (Figures 2C,H).

### The Absence of SO Confers Reduced Biomass Accumulation in *Arabidopsis* Plants Exposed to Carbon Starvation

**Figure 3:**
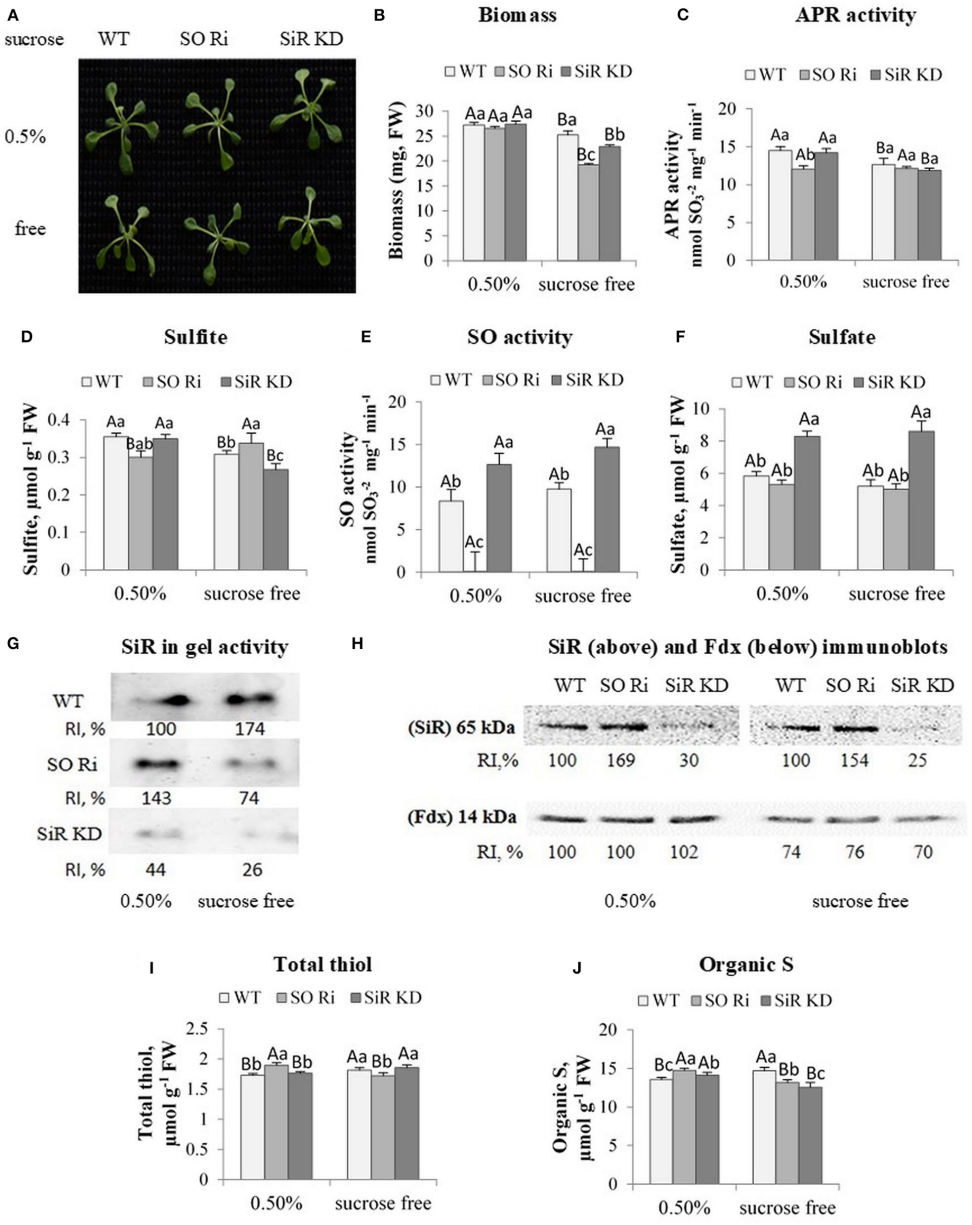
Characterization of *Arabidopsis* wild-type (WT), as well as sulfite reductase (SiR KD) and sulfite oxidase (SO Ri), modified plants in response to 0.5% (normal) and 0% sucrose (sucrose-free) conditions. **(A)** Plants appearance, photographed 9 d after transfer, of 8-day-old seedlings, to agar plates containing 0.5 MS with or without sucrose. **(B)** Total biomass accumulation of the upper plants part. Values are means (*n* = 20 plants). **(C)** APR activity. **(D)** Sulfite level. **(E)** Activity of SO enzyme assayed as sulfite disappearance. **(F)** Sulfate level, **(G)**. SiR in-gel activity indicated by the appearance of sulfide, the final product of SiR activity reacted with lead acetate to generate the brown precipitate of lead sulfide. Crude extracts of carbon treated plants were fractionation employing NATIVE PAGE conditions with two separated gels with control plants (see the full gels in Figure 4D). RI represents relative intensity (%) compared with the activity of WT treated with 0.5% sucrose as 100%. **(H)** Immunoblot analyses of *Arabidopsis* SiR (upper insert) and Fd (lower insert) proteins fractionated by SDS-PAGE and immunoblotted with SiR- and Fd-specific antiserums. Each lane contains 10 μg of proteins for SiR and 30 μg for Fd. Relative intensity, % (RI, %). **(I)** Total non-protein thiol and **(J)**. Organic S level. The organic S was calculated as the difference between total S to the inorganic S (sulfate + sulfite). Values for **(C–F,I,J)** represent the means of three independent experiments (±SE, *n* = 3). **(G,H)** Represent one of three independent experiments with similar results. Different lowercase letters indicate differences between genotypes within the same treatment. Different uppercase letters indicate significant differences within the plant genotypes in response to treatment (Tukey–Kramer HSD test; JMP 8.0 software).

Eight-day-old WT, SO Ri, and SiR KD seedlings were transferred to grow in the growth room for 9 days in 0.5 MS agar plated containing normal (0.5% sucrose) and starved (sucrose free) as the carbon source to study the role of SO in carbon metabolism. The various sucrose concentrations in the growth medium may affect the osmotic potential and its effect on the carbon source level. Although the measurement of the osmotic potential of the 0.5 MS media supplemented with and without sucrose revealed 126± 0.7 and 123 ± 1.5 mmol/kg, respectively. However, the plants grown in the higher osmotic potential containing the 0.5% sucrose exhibited better performance [higher biomass accumulation (Figures 3A,B)], indicating that the carbon rather than the high osmoticum affected the plants.

Notably, carbon starvation led to a lower biomass accumulation rate in SO, and SiR impaired plants than WT, whereas plants grown in the presence of 0.5% sucrose did not show any differences in shoot biomass between genotypes (Figures 3A,B). APR activity was detected to examine if the lower biomass accumulation in mutant plants is due to the inability of the mutants to detoxify sulfite generated by the enzyme. Notably, an inspection of APR activity revealed a reduction under sucrose starvation in WT and SiR KD compared with the control-treated plants, whereas the lowest APR activity in SO Ri among the control-treated genotypes was not affected by sucrose starvation (Figure 3C). Measurement of sulfite level followed APR activity showed a decreased level in SiR KD and WT, but not in SO Ri that was increased at the absence of sucrose (Figure 3D), despite the unchanged APR activity (Figures 3C,D), indicating reduced sulfite detoxification by oxidation in the absence of active SO.

Notably, the smaller biomass reduction in WT and SiR KD compared with SO Ri grown without sucrose (Figure 3B) indicates that KD mutant plants were less stressed than SO Ri plants. This is most likely due to active sulfite oxidase in SiR KD mutant plants, essential to detoxify by oxidation the generated sulfite. Indeed, SO activity in SiR KD was higher than in WT under both sucrose level conditions (Figure 3E), and such detoxification of sulfite was recently shown to be less metabolically costly than the reduction to sulfide by SiR activity, avoiding the futile cycle of reduction and degradation (Yarmolinsky et al., 2013). In support of the rate of detoxification by SO oxidation activity, the significantly higher sulfite level in SO Ri grew under carbon deficiency than plants grown under normal (0.5%) growth conditions. In contrast, in SiR KD and WT, a sulfite decrease was noticed (Figure 3D) due to enhanced SO activity (Figure 3E). Additionally, a significant 1.5-fold higher accumulation rate of sulfate was evident in SiR KD mutant than WT and SO Ri plants grown under both sucrose treatments (Figure 3F). The higher accumulation rate of sulfate in SiR impaired plants contributed to the significantly higher total S content under both sucrose treatments (Supplementary Figure 2).

### Impairment of SO Led to a Reduced Sulfur Reduction Pathway Under Sucrose Depletion

**Figure 4:**
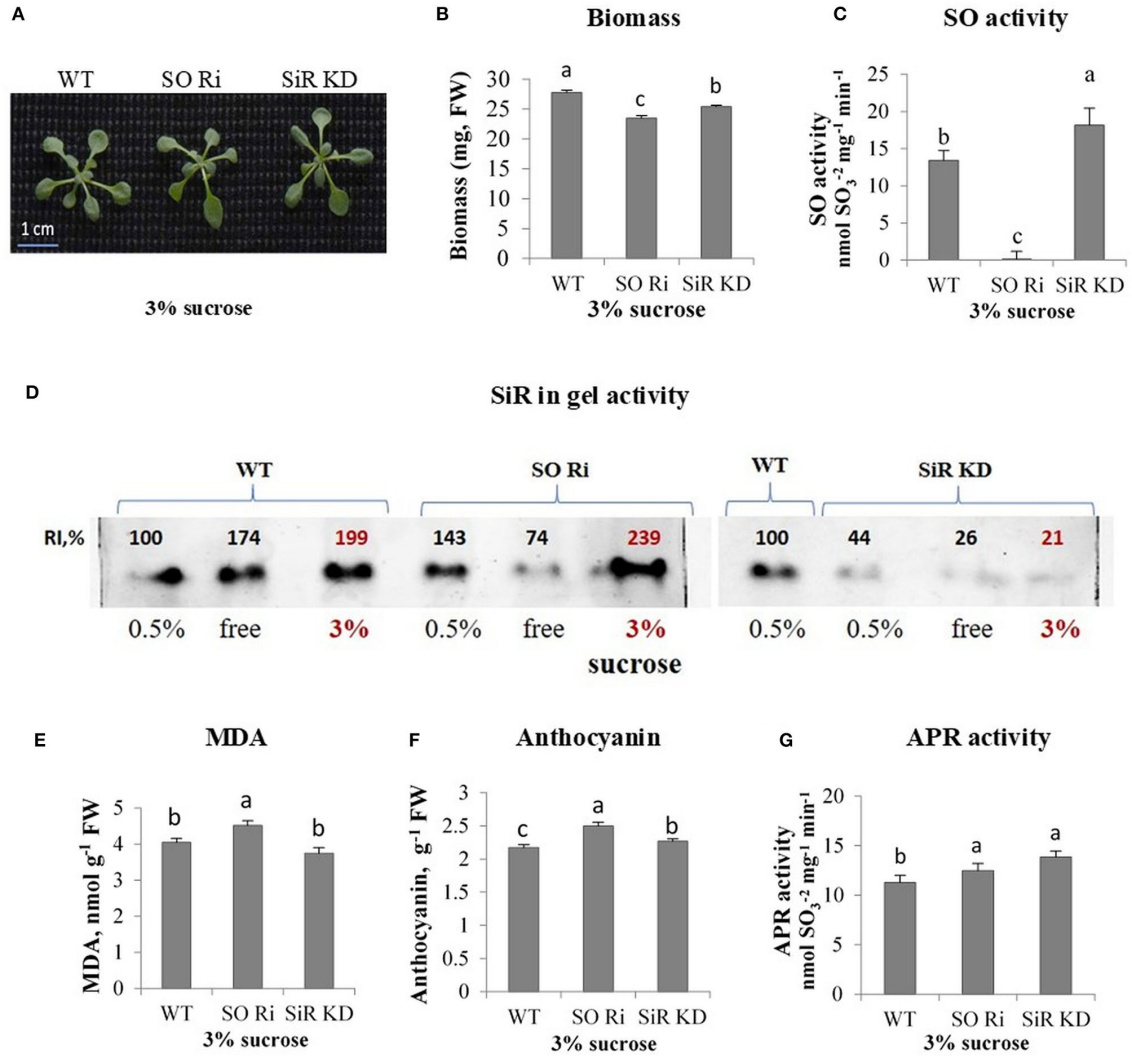
Characterization of *Arabidopsis* wild-type (WT) and sulfite reductase (SiR KD), sulfite oxidase (SO Ri) modified plants in response to excess (3% sucrose) carbon in the growth medium. **(A)** Plants appearance, photographed 9 d after transfer, of 8-day-old seedlings, to agar plates containing 0.5 MS with or without sucrose. **(B)** Total biomass accumulation of the upper plants part. Values are means (*n* = 20 plants). **(C)** SO activity assayed as sulfite disappearance. **(D)** SiR in-gel activity indicated by the appearance of sulfide, the final product of SiR activity, reacting with lead acetate to generate the brown precipitated activity band of lead sulfide. Crude extracts of carbon treated plants (as 3% sucrose, bolded red font is used to emphasize those points) were subjected to electrophoretic gel fractionation under native conditions. The two separated gels with three (0.5%, free, and 3% sucrose) were assayed together simultaneously. Since the 3% sucrose-treated WT was with ca. 2-fold higher intensity than 0.5% treated WT as the control, and the 3% sucrose treated SiR KD was more than 4-fold lower than the 0.5% sucrose treated WT as the control of the right INSERTED gel, then SiR KD treated with 3% was significantly lower as compared with WT and SO RI treated with 3% sucrose in the left inserted gel. **(E)** Malondialdehyde (MDA) level. **(F)** Anthocyanin level. **(G)** APR activity rate. Values in **(C–G)** represent one of three independent experiments with similar results (±SE, *n* = 3). Different lowercase letters indicate differences between genotypes within a treatment (Tukey–Kramer HSD test; JMP 8.0 software).

Sulfite oxidase and SiR have been characterized as essential players in plant protection against sulfite toxicity, demonstrating that mutants impaired in these activities were significantly damaged by toxic sulfite application (Brychkova et al., 2007; Lang et al., 2007; Yarmolinsky et al., 2013). Notably, SiR activity was increased in SO Ri under control [0.5% sucrose (left activity band in three inserts, Figure 3G)] and was decreased in plants grown without sucrose in the growth medium [Figure 3G, middle insert (full activity gels is presented in Figure 4D)], likely resulting in the sulfite increase in sucrose starved SO Ri (Figure 3D). While SiR activity rate was much lower in SiR KD than in WT control plants, SiR KD exhibited almost undetectable SiR activity compared with the highest in-gel activity of WT sucrose starved plants (Figure 3G, lowest insert). Western blot analyses of SiR protein showed higher expression levels than in WT leaves in SO Ri mutant grown with and without sucrose (Figure 3H, upper insert), indicating a sulfur starvation response in SO Ri as the result of carbon starvation. Notably, ferredoxin (Fdx) protein that acts as the physiological donor of the six electrons required for sulfite reduction by SiR (Nakayama et al., 2000; Yonekura-Sakakibara et al., 2000) exhibited significantly lower expression levels in response to sucrose deficiency in all genotypes, compared with 0.5% sucrose (Figure 3H, lower insert), supporting the notion that the decrease in Fdx expression level, the result of carbon starvation is likely the cause for SiR activity decrease resulting in the sulfite enhancement in carbon starved SO Ri. Unlike the carbon-starved mutant, WT plants exhibited SiR-enhanced activity, resulting in a more efficient system of sulfite detoxification by both SO and SiR, resulting from the absence of futile sulfite oxidation or reduction cycles.

The sulfur starvation response noticed by the highest expression level of SiR protein in contrast to the lower SiR activity rates in SO Ri mutants grown under carbon starvation is supported by the decrease in total thiol levels compared with SiR KD and WT, while the highest SiR activity rate in SO Ri grown under normal carbon supply led to significantly higher thiol (Figure 3I) as well as enhanced organic-S accumulation rate (Figure 3J). Notably, under normal carbon supply, the lower organic S level in WT and SiR KD compared with SO Ri mutant was sufficient to accumulate similar biomass among the three genotypes. In contrast, carbon starvation resulted in lower organic S levels in both mutants than WT, followed by a significantly lower biomass accumulation rate than WT plants (Figures 3B,J). These results indicate that active SiR and active SO are essential for the interaction of sulfur and carbon metabolism, especially under carbon starvation.

Like WT, yet with higher total thiol levels than SO Ri, while being the lowest in organic S level compared with WT. SO Ri leaves in plants exposed to carbon starvation is likely the consequence of alternative sulfite detoxification pathways when SiR is blocked, and excess sulfite is accumulated, such as the synthesis of the less toxic thiosulfate by STs (Tsakraklides et al., 2002; Papenbrock et al., 2011), as shown before with tomato SiR KD mutants (Yarmolinsky et al., 2014). Moreover, this highlights the role of STs in detoxification of sulfite to sulfide when SiR is blocked and after that in filling up the thiols pool (see above, Figure 2H).

### Impairment in SO and SiR Led to Reduced Biomass Accumulation in Plants Grown on Excess Carbon Supply

The sucrose used as a carbon source in the 0.5 MS culture media may act as an osmotic agent at certain concentrations. Despite the increasing concentration of sucrose in MS media to 3%, biomass accumulation of WT did not change compared with plants grown under normal carbon supply [0.5% sucrose (Supplementary Figures 3A,B)]. In contrast, excess sucrose supply (3% sucrose) reduced total plant biomass in both SO Ri and SiR KD compared with WT, showing the highest biomass decrease in SO Ri (Figures 4A,B). Notably, the impairment in SiR was followed by a rise in SO activity rate compared with WT (Figure 4C) to compensate for the partial absence of normal SiR activity as shown before (Yarmolinsky et al., 2013). Similarly, the impairment in SO was followed by an increase in SiR activity (Figure 4D). As expected, the enhanced SiR activity in impaired SO plants, the result of the higher accumulated sulfite, resulted in higher water-soluble thiol levels compared with WT and SiR KD (Table 1). Measurement of sulfate revealed a 1.6-fold enhanced accumulation, resulting in enhanced SO activity in SiR KD mutants, as compared with WT (Table 1). No differences in detected sulfate levels between WT and SO Ri were noticed (Table 1), likely the result of sulfite oxidation in SO Ri by reactive oxygen species (ROS; Hansch et al., 2006; Brychkova et al., 2012a). The decrease in total glutathione in SO and SiR impaired plants is most likely due to oxidative stress-induced consumption of the glutathione (Table 1), which is a donor of reducing equivalents for ROS scavenging (Noctor and Foyer, 1998). This is supported by the increase in the levels of the oxidative stress markers; the enhancement of MDA in SO Ri mutants compared with WT, and the increased level of the antioxidants, anthocyanins (Gould et al., 2002; Tian et al., 2017; Xu et al., 2017) in both mutants compared with WT (Figures 4E,F). In addition, enhanced APR activity in both mutants compared with WT is likely due to oxidative stress induction (Figures 4E–G) shown before to result in APR induction (Bick et al., 2001).

**Table 1:** Levels of sulfite, sulfate, total glutathione, total thiol, total sulfur, and organic detected in *Arabidopsis* WT, SO Ri, and SiR KD plants are grown on MS media containing 3% sucrose.

**Genotype ^a^**	**Sulfite^b^**	**Sulfate^c^**	**Total glutathione^d^**	**Total Sulfur^e^**	**Total thiol^f^**	**Organic S^g^**
	μmol g^−1^ fresh weight					
WT	0.32 ± 0.04^b^	6.72 ± 0.32^b^	0.46 ± 0.003^a^	24.9 ± 0.1^b^	2.42 ± 0.04^b^	17.8 ± 0.03^b^
SO Ri	0.4 ± 0.02^a^	7.98 ± 0.54^a^^b^	0.42 ± 0.002^b^	24.08 ± 0.6^b^	2.73 ± 0.02^a^	15.7 ± 0.02^c^
SiR KD	0.31 ± 0.01^b^	8.36 ± 0.77^a^	0.42 ± 0.003^b^	28.33 ± 0.2^a^	2.01 ± 0.04^c^	20.2 ± 0.01^a^

*Different lowercase letters show significant differences between the genotypes within treatment, as calculated using Tukey–Kramer HSD test JMP8.0 software (http://www.jmp.com/)*.

a *The detected metabolite concentrations data are means of n = 3 SE for WT, SO Ri, and SiR KD. The values were normalized by the dry weight content of WT grown under the same 3% sucrose condition and expressed in fresh weight.*

b *Sulfite was detected by a specific sulfite detection assay using chicken SO (Brychkova et al., 2012a).*

c *Sulfate was detected by Ion chromatography, as described in material and methods.*

d *Total glutathione was determined according to Griffith (1980) as described in material and methods.*

e *Total sulfur was measured by inductively coupled plasma emission spectrometry (ICP-AES), as described in material methods.*

f *Total thiol was detected using DTNB as described by De Kok et al. (1997).*

g *The organic S was calculated as the difference between total S to the inorganic S (sulfate + sulfite)*.

The total sulfur content of WT and SO Ri generally followed the sulfate levels, being similar between WT and SO Ri, while partial blocking of sulfite reduction led to increasing sulfite oxidation by SO and enhanced total S in SiR KD mutant (Table 1). Interestingly, the excess carbon condition resulted in the highest thiols level and the lowest organic-S in SO Ri plants. In contrast, SiR KD exhibited the lowest thiols and highest organic-S levels (Table 1). Considering that both mutants accumulated lower biomass than WT grown under carbon (Figure 4B), these results indicate that optimal sulfur metabolism is essential to cope with carbon stress. The high level of MDA and anthocyanin and the lower total glutathione in the mutants compared with WT (Figures 4E,F, Table 1) suggest induction of oxidative stress that resulted in the lower growth and biomass accumulation in the mutants.

## Discussion

### The Absence of Activity of SO Reduced Biomass Accumulation in Plants Exposed to the Sulfate Treatment

**Figure 5:**
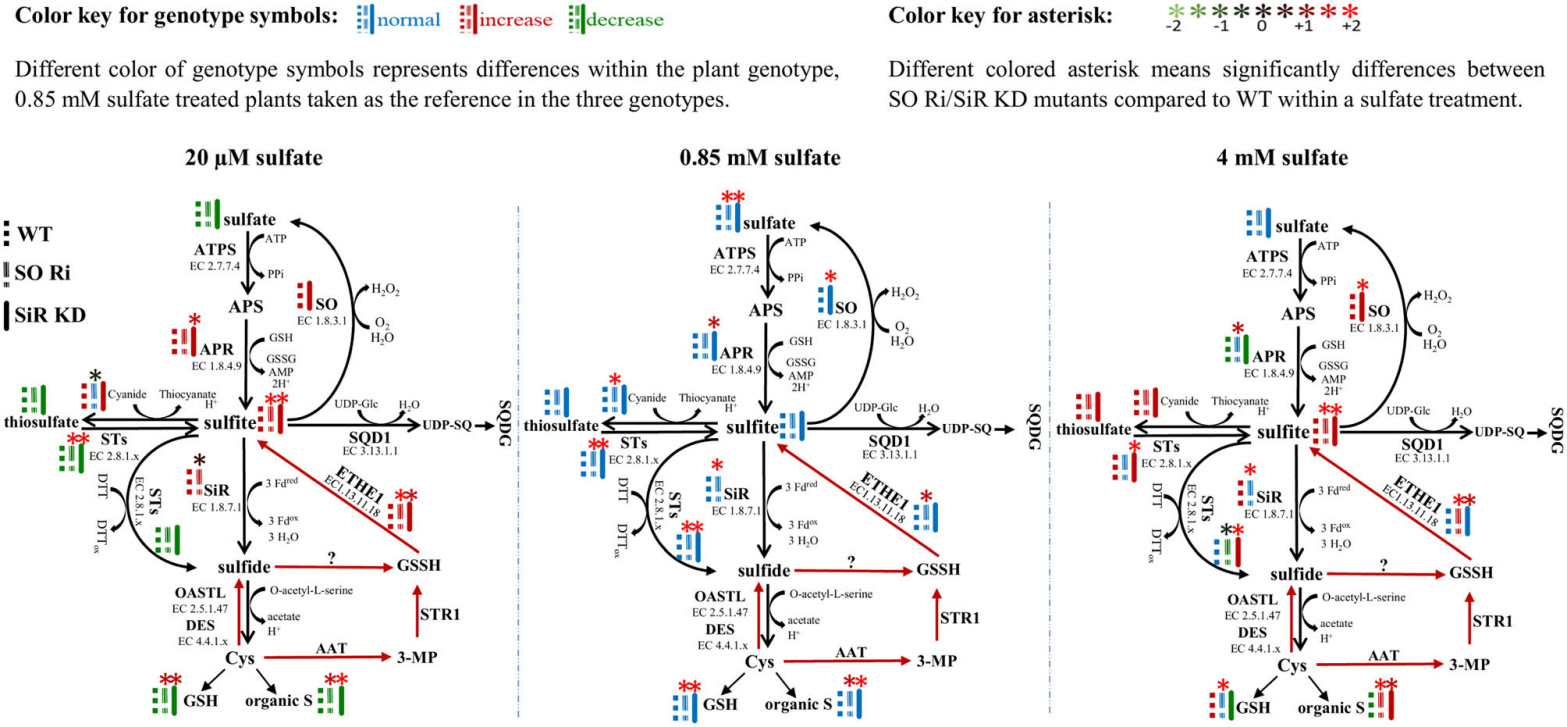
Schematic illustration describing the impact of sulfite oxidase (SO) and sulfite reductase (SiR) impairment on the sulfur metabolism in *Arabidopsis* plants grown under sulfate starvation (20 μM), normal sulfate (0.85 mM), and excess sulfate (4 mM) levels as the only S source in 0.5 MS medium. Different color of genotype symbols represents differences within the plant genotype, 0.85 mM sulfate treated plants taken as the reference in the three genotypes (see above color key). Different colored asterisk means significant differences between SO Ri/SiR KD mutants compared with WT within a sulfate treatment (see above color key). The presented significant differences are based on statistical analyses shown in Figures 1, 2 and Supplemental Figure 1. The organic S was calculated as the difference between total S to the inorganic S (sulfate + sulfite). Red arrows indicate Cys catabolic pathway (Krüßel et al., 2014). ATPS, adenosine phosphate sulfurylase; APS, adenosine-5′-phosphosulfate; APR, APS reductase; SiR, sulfite reductase; STs, sulfur transferases; SQD1, UDP-sulfoquinovose synthase1; UDP-SQ, UDP-sulfoquinovose; SQDG, sulfolipid 6-sulfo-α-d-quinovosyl diacylglycerol; OAS-TL, O-acetyl-serine-thiol-lyase; SO, sulfite oxidase; Cys, cysteine; GSH, reduced glutathione; GSSG, oxidized glutathione; GSSH, glutathione persulfide; ETHE1, ethylmalonic encephalopathy protein1; DES, L-cysteine desulfhydrase; Fdox, Oxidized ferredoxin; Fdred, reduced ferredoxin; H_2_O_2_, hydrogen peroxide; 3-MP, 3-mercaptopyruvate; AAT, aminotransferase; PPi, diphosphate; DTT, dithiothreitol; DTTox, Oxidized dithiothreitol.

The application of different sulfate level treatments demonstrated that SO impairment significantly affects sulfur metabolism, resulting in lower biomass and high accumulation of sulfite and reduced sulfur compounds under non-optimal sulfate supply conditions, resulting in enhanced sulfite level (Figure 5, left and right inserts). In contrast, WT, SO Ri, and SiR KD mutant plants are grown under optimal sulfate level exhibited a normal sulfate reduction pathway and (Figure 5, middle insert) did not differ in their accumulated biomass. Among the sulfate starved or excess sulfate treated plants, SO Ri showed a significant reduction in total plant biomass accumulation. In contrast, SiR KD mutant showed similar biomass accumulation as wild type (Figure 1B). This suggests that KD plants were more tolerant of starvation and excess sulfate conditions than SO Ri plants. However, SO Ri and SiR KD mutant plants accumulated the same high sulfite levels as WT under starvation and excess sulfate growth conditions (Figure 5), pointing to the importance of each enzyme's role in sulfite utilization. The absence of differences in biomass accumulation between WT and SiR modified plants grown under starvation, and excess sulfate might indicate sufficient sulfite detoxification by the active SO (Brychkova et al., 2007, 2013; Lang et al., 2007) in SiR KD mutant plants. An increase in SO's less metabolically costly activity was observed in WT and SiR KD mutant plants, being ~1.2- and 1.7-fold higher under starvation and excess sulfate, respectively, compared with WT under normal conditions. [0.85 mM sulfate (Figure 1C)]. This indicates that SO acts to avoid sulfite toxicity as a safety valve, maintaining a homeostatic sulfite level in plant tissue. A similar response of sulfite consumption by STs under sulfur starvation and excess sulfate treatments was observed in all three genotypes, except for SO Ri under sulfate starvation (Figure 2D). However, the enhancement of SO and STs in sulfite oxidation and consumption activities, respectively, under starvation or excess sulfate relative to normal growth conditions, was not enough to equalize sulfite level in SiR KD to WT plants (Figure 5). This highlights the importance of sulfite reductase in the detoxification of sulfite. Indeed, the absence of SO led to an enhancement of SiR activity under all sulfate applications (Figure 5) and a high accumulation rate of GSH, total thiol compounds, and organic S under starvation and excess sulfate conditions (Figure 5, Supplementary Figure 1A). These results indicate that the absence of SO activity leads to an imbalance in sulfite homeostasis, which is compensated for by a metabolic shift toward a metabolically costly sulfite reduction pathway, leading to elevated levels of reduced sulfur-type compounds.

Notably, total thiol and organic-S levels significantly increased under excess sulfate application in SiR KD, as compared with WT (Supplementary Figure 1A, Figure 5), contrasting with the assumption that organic S is downregulated in SiR impaired plants as a result of lower SiR activity rate (Khan et al., 2010). Taking into consideration the significantly higher activity rate of sulfide production by STs in SiR KD mutant plants under excess sulfate (**Figures 2D–H, 5**, right insert), this points to an efficient sulfite to sulfide detoxification mechanism by STs, which results in a significantly higher accumulation rate of reduced S. A possible role in sulfide biogenesis through interaction with reductants such as thioredoxin was suggested for STs such as STR1, STR14, STR15, and STR16 (Balmer et al., 2004; Yoshida et al., 2013; Henne et al., 2015). These results imply that the absence of SiR uncovers possible roles for STs in sulfite detoxification and the production of sulfide in plants.

### Various Sulfite Generation Activities Contribute to Sulfite Levels in *Arabidopsis*

Sulfite accumulation in plants is the consequence of sulfite generation rate by enzymes such as APRs, STs, or ETHE1 (Kopriva and Koprivova, 2004; Papenbrock et al., 2011; Brychkova et al., 2013; Krüßel et al., 2014) and the utilization by the sulfite network enzymes such as SO, SiR, STs and sulfoquinovosyldiacylglycerol 1 (SQD1) (Sanda et al., 2001; Lang et al., 2007; Khan et al., 2010; Papenbrock et al., 2011; Brychkova et al., 2013; Yarmolinsky et al., 2013). Notably, sulfite up-regulation was noticed in all genotypes grown under sulfate starvation and excess sulfate compared with the normal sulfate condition (Figure 5). The high accumulation rate of sulfite under 20 μM sulfate is likely the result of APR activity (Figures 1E, 5, left insert) caused by sulfur deficiency and by oxidative stress (Bick et al., 2001; Hirai et al., 2003; Koprivova et al., 2008), indicated by the high accumulation rate of MDA and antioxidant anthocyanins (Figures 1F,G). However, plants with a lower APR activity rate accumulated the same level of sulfite under 4 mM as compared with 20 μM sulfate (Figure 5, left and right inserts), indicating additional source/s for sulfite generation.

The enhancement of metabolites such as GSH and total thiols in SO Ri and SiR KD compared with WT leaves in plants grown with excess sulfate (Figure 1H, Supplementary Figure 1A), down-regulated APR activity (Figure 1E), indicating that the accumulated sulfite can be caused either by STs using thiosulfate as the substrate (Papenbrock et al., 2011; Brychkova et al., 2013) or by ETHE1 oxidizing GSSH to sulfite (Krüßel et al., 2014). Indeed, an increase of sulfite-producing activity rate by STs and the net STs activity in favor of sulfite generation rather than consumption was noticed in all genotypes except SO Ri has grown with excess sulfate (Figures 2F,G, 5, right insert).

In plants, generated sulfide can be non-enzymatically oxidized to GSSH in the presence of GSSG (Rohwerder and Sand, 2003; Krüßel et al., 2014). GSSH can also be generated by STR1 employing β-mercaptopyruvate and GSH (Höfler et al., 2016), and the resulting GSSH can be oxidized to sulfite by ETHE1 as shown in *Arabidopsis* (Krüßel et al., 2014). Notably, the ETHE1 transcript exhibited higher relative expression in all genotypes under the lowest sulfate than normal and excess sulfate applications (Supplementary Figure 1C). This alteration under the lowest sulfate might indicate a rebalancing of the resources in adaptation to a nutrient limitation and indicate that accumulated sulfite under the lowest sulfate supply was generated from both APR and ETHE1. A significant 3- and 1.8-fold increase in the expression of ETHE1 transcript in SO Ri and SiR KD, respectively, compared with WT under excess sulfate supply indicate that the source of sulfite can be the consequence of ETHE1 activity, the recycling result of the high accumulated thiols (Supplementary Figures 1A,C). This notion is supported by the significantly high activity rate of sulfide production in SO Ri and SiR KD by SiR and STs activities, respectively, as well as the enhanced GSSG accumulated in the mutants compared with WT grown with high sulfate (Figure 5, right insert, Supplementary Figure 1B).

Interestingly, a higher expression rate of ETHE1 transcript was noticed in SO Ri compared with WT, and SiR KD grew in the three different sulfate levels supplied (Supplementary Figure 1C). This likely indicates that in the absence of active SO, the futile recycling of the product of sulfite already entered into the sulfate reduction pathway and was being released again as a result of sulfur-containing metabolite turnover (Tsakraklides et al., 2002), highlighting the role of SO in the optimal sulfur metabolism. Additionally, these results indicate that APR, ETHE1, and STs are potential sources for sulfite accumulation in SO Ri and SiR KD plants grown under the lowest and excess sulfate.

### Low and High Carbon in the Growth Medium Implies Reduced Biomass Followed by Sulfite Accumulation in *Arabidopsis* SO Ri Impaired Plants

**Figure 6:**
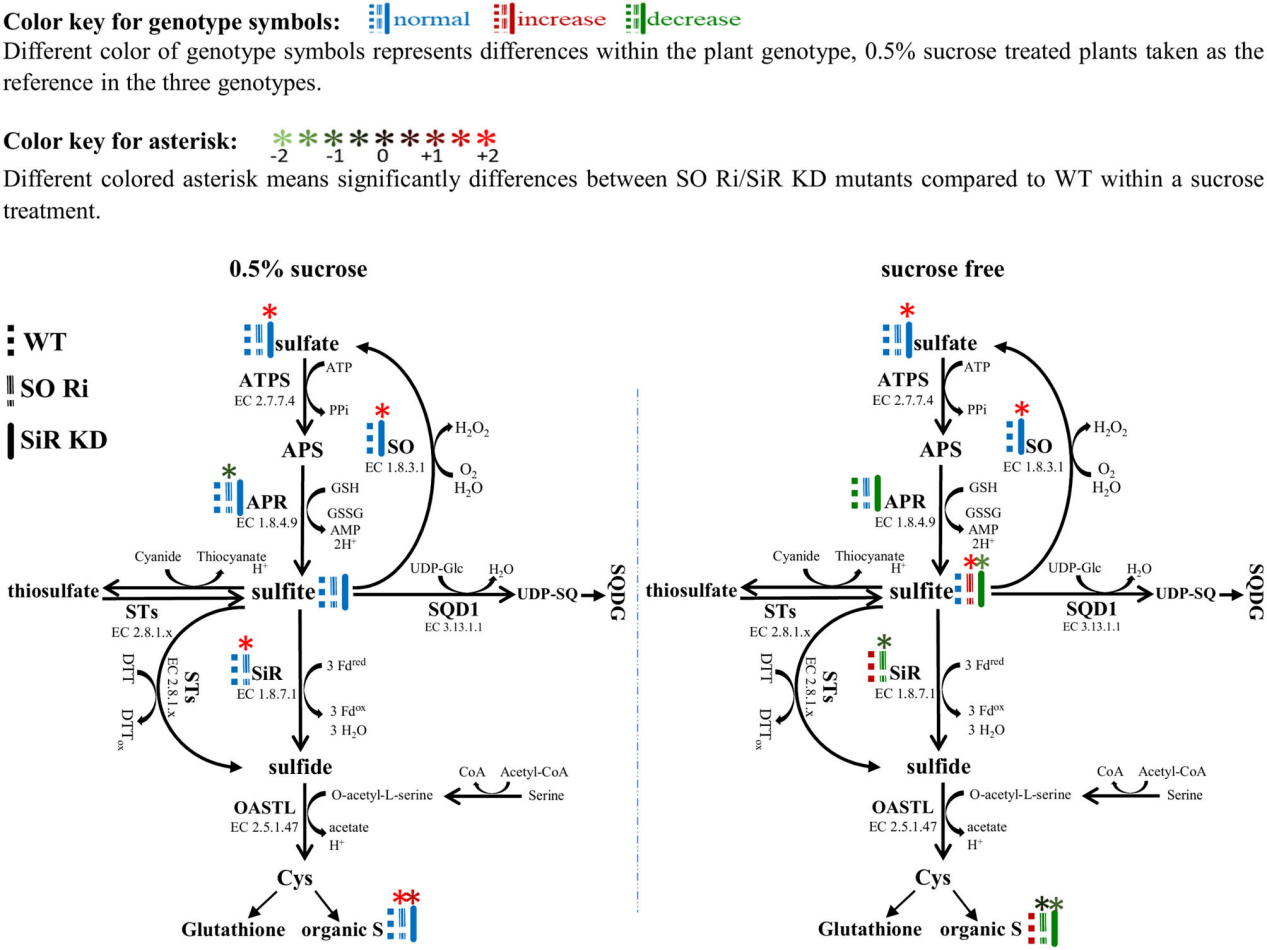
Schematic illustration depicting the impact of sulfite oxidase (SO) and sulfite reductase (SiR) impairment on the sulfur metabolism in *Arabidopsis* plants grown under normal (0.5% sucrose) and sucrose starvation (sucrose free) conditions. Genotype symbols with different colors represent differences within the plant genotype; 0.5% sucrose-treated plants are taken as the reference in the three genotypes (see above color key). All of the presented significant differences are based on statistical analyses shown in Figure 3. The organic S was calculated as the difference between total S to the inorganic S (sulfate + sulfite). ATPS, adenosine phosphate sulfurylase; APS, adenosine-5′-phosphosulfate; APR, APS reductase; SiR, sulfite reductase; SO, sulfite oxidase; STs, sulfur transferases; SQD1, UDP-sulfoquinovose synthase1; UDP-SQ, UDP-sulfoquinovose; SQDG, sulfolipid 6-sulfo-α-d-quinovosyl diacylglycerol; OAS-TL, O-acetyl-serine-thiol-lyase; Cys, cysteine; GSH, reduced glutathione; Fd_ox_, Oxidized ferredoxin; Fd_red_, reduced ferredoxin; H_2_O_2_, hydrogen peroxide; PPi, diphosphate; DTT, dithiothreitol; DTT_ox_, Oxidized dithiothreitol.

The activity of APR, the key enzyme of the sulfate reduction pathway, was shown to be subjected to metabolic control by carbon metabolism, exhibiting enhancement with carbon source increase (Hesse et al., 2003). The response of SiR, which functions downstream to APR in the pathway and SO to carbon level, was rarely shown in *Arabidopsis*. Growing *Arabidopsis* WT, SO Ri, and SiR KD mutant plants for 9 days in a carbon-free MS medium led to a significantly reduced biomass accumulation rate in all the genotypes, compared with the control plants supplied with 0.5% sucrose in the growth medium. Notably, in the carbon starved plants, the highest biomass level was noticed in WT and the lowest in SO Ri plants (Figures 3A,B). The latter is attributed to the high sulfite accumulation, the consequence of the imbalance between sulfite generation by APR and its utilization by SiR. Accordingly, a decrease in APR activity rate was evident in WT and SiR KD under sucrose starvation, whereas in SO Ri, APR activity did not vary compared with the sufficient carbon under control conditions (Figures 3C, 6). The enhanced sulfite level while APR activity was not enhanced in SO Ri under carbon starvation compared with SO Ri grown on the control medium is the result of SiR activity decrease under carbon starvation, although SiR protein in SO Ri was enhanced as compared with WT in both growth conditions: control and carbon starvation (Figures 3G,H, upper insert, **6**). In support of this notion is the lower accumulation rate of thiols, organic and total S noticed in carbon starved SO Ri compared with WT plants under carbon starvation (Figures 3I,J, Supplementary Figure 2), indicating less efficient sulfur assimilation in plants with inactive SO under carbon limitation. Since to catalyze the reduction of sulfite to sulfide, the SiR enzyme requires ferredoxin as the electron donor (Nakayama et al., 2000; Yonekura-Sakakibara et al., 2000), the ferredoxin expression was detected, and it was revealed that ferredoxin protein expression was significantly lower in response to sucrose deficiency in all genotypes, compared with the 0.5% sucrose treatment (Figure 3H, lower insert). This indicated that the high SiR protein expression level and the drop of ferredoxin-dependent sulfite reductase in SO Ri were presumably due to an energy deficiency caused by the lack of enough carbon (Figures 3G,H, upper insert) exhausted by the futile sulfite reduction of excess sulfite *via* SiR activity. Interestingly, despite the lower expression level of ferredoxin protein, WT plants exhibited enhanced SiR activity, resulting in a more efficient system of sulfite detoxification by both SO and SiR and the absence of metabolically costly sulfite reduction, as compared with carbon starved SO Ri mutants.

Overall, these results demonstrate that existing active SiR in SO Ri mutant plants is insufficient to maintain optimal sulfite homeostasis under reduced carbon conditions, highlighting the efficiency of sulfite oxidase in sulfite detoxification under carbon starvation. Our results indicate that SO activity is essential to maintain sulfur metabolism in carbon-stressed plants.

Subjecting SO Ri mutant plants to growth conditions containing excess carbon resulted in a reduction in total biomass, total glutathione, and organic S, together with increased sulfite, MDA, and antioxidant anthocyanins compared with WT plants (Table 1, Figures 4A,E,F, Supplementary Figure 4). Since SiR was shown to efficiently detoxify sulfite in *Arabidopsis* and tomato (Yarmolinsky et al., 2013, 2014), it is reasonable to expect a role of SiR in the utilization of excess sulfite in the absence of active SO enzyme in the SO Ri mutants. However, the 40% higher SiR activity rate than WT was insufficient to maintain sulfite levels as in the other genotypes examined (Table 1, Supplementary Figure 4). The decrease in biomass of SO Ri is not necessarily a result of direct sulfite toxicity since no visible damage was noticed in leaves (Figure 4A). Sulfite enhancement was already shown to induce ROS generation by NADPH oxidase and other FAD-containing enzymes in plant leaves (Brychkova et al., 2012a; Yarmolinsky et al., 2014). The lower total glutathione and the higher MDA levels, the result of lipid peroxidation, as well as the enhanced anthocyanin, shown as potent antioxidants (Yamasaki et al., 1996; Wang et al., 1997) further support the notion of sulfite induced oxidative stress that resulted in the lower biomass accumulation rate.

Notably, SiR KD mutant plants exhibited lower biomass accumulation rates than WT but higher than SO Ri, yet with similar sulfite accumulation as WT despite the enhanced APR activity among the genotypes tested (Figures 4A,B,G, Table 1, Supplementary Figure 4). These results indicate the efficiency of the enhanced SO activity in SiR KD mutant to oxidize the excess sulfite to the highest sulfate level detected in SiR KD, decreasing the oxidative stress level as indicated by the similar MDA level, albeit with enhanced anthocyanin as compared with WT (Figures 4C,E,F, Supplementary Figure 4). Overall, these results indicate that SO an expression is an essential tool for regulating toxic sulfite during sulfate assimilation and properly controlling sulfite homeostasis to avoid oxidative stress under excess carbon treatment.

## Conclusion

By employing *Arabidopsis* WT, SiR, and SO impaired plants exposed to excess and limited carbon or sulfate levels, the important role of SO in carbon and sulfur metabolism is demonstrated. Application of limited or excess sucrose or sulfate levels led to sulfite increase that affected biomass accumulation in the mutant compared with WT plants. SO-impairment led to the channeling of the excess sulfite toward the sulfur reduction pathway, resulting in futile consumption of reductant/energy and accumulation of organic S. Additionally, the enhancement of sulfite resulted in oxidative stress that contributed as well to the decrease in biomass accumulation. These results indicate that the role of SO is not limited to protection against elevated sulfite toxicity but to maintaining optimal carbon and sulfur metabolism in *Arabidopsis* plants.

## Materials and Methods

### Plant Material

In the present study, we used *Arabidopsis* thaliana plants (ecotype Columbia, WT), SiR knockdown (KD) (SALK075776) mutant line as described by Yarmolinsky et al. (2013), and SO RNA interference (Ri) lines as described by Brychkova et al. (2007).

### Carbon Treatment

Starvation condition in MS medium was obtained by omitting sucrose from the MS medium. Eight-day-old seedlings of WT and SiR or SO modified plants were transferred from 0.6% agar plates onto 0.5% MS supplemented with 1% agar plates in the presence (0.5%), absence (sucrose free), or excess (3%) sucrose. Each Petri dish contained five plants grown for 9 days. The 17-day-old plants grown on sucrose, sucrose free, and 3% sucrose (0.5 MS media) were harvested, weighed, and frozen immediately to monitor the changes under carbon starvation. Plants were grown in a growth room under 10 h light/14 h darkness, 22°C, 75–85% relative humidity, and 150 μmol m^−2^ s^−1.^

### Sulfate Treatment

Plants were exposed to sufficient (0.85 mM), starvation (20 μM), or excess (4 mM) sulfur content (as sulfate) in the growth medium to examine the performance of WT and SiR, SO impaired plants under vast S conditions in terms of biomass production, metabolite level, and enzymes activity. Eight-day-old seedlings of WT and SiR or SO modified plants were transferred from 0.6% agar plates onto 0.5% MS agar plates containing 20 μM, 0.85 mM, or 4 mM sulfate as the only source of sulfur and grown for 9 days. To monitor the changes in genotypes under three levels of sulfate treatment, the 17-day old plants were harvested, weighed, and frozen immediately.

### Immunoblotting Analysis

Proteins for SiR and Fdx were extracted in buffer containing 250 mM Tris-HCl (pH 8.48), 1.25 mM EDTA, 14 mM GSH, 4 mM dithiothreitol, 5 mM L-Cys, 0.5 mM sodium molybdate, 250 mM Suc and a cocktail of protease inhibitors (https://www.sigmaaldrich.com) including aprotinin (10 μg ml^−1^), leupeptin (10 μg ml^−1^) and pepstatin (10 μg ml^−1^) and separated in 12.5% (w/v) acrylamide gels by SDS-PAGE followed by transfer to polyvinylidene difluoride membranes and immunoblotted with SiR and Fdx-specific antiserums as described before (Brychkova et al., 2007; Yarmolinsky et al., 2014). Lanes were loaded either with 10 μg of protein (for SiR) or 30 μg (Fdx). Protein bands were visualized by staining with the enhanced chemiluminescence Super Signal Western Blotting System (Pierce; http://www.piercenet.com).

### Direct in-Gel SiR Activity

In-gel SiR activity was detected using our lab protocol of H_2_S in-gel visualization (Brychkova et al., 2012c). The reaction solution contained 0.05 M Tris–HCl, pH 7.5, 50 mM β-mercaptoethanol, 6 mM sodium dithionite (dissolved in 150 mM NaHCO_3_), 0.7 mM MV, 0.15 mM NADPH (unless otherwise specified) and 0.4 mM lead acetate. When required, the reaction time was extended by adding a fresh reaction solution. The reaction was stopped by immersion of the gel in double-distilled water.

### Protein Extraction and Kinetic Assays for SO, APR, and ST Activities

Protein extraction and activities of SO and APR were performed as described before (Brychkova et al., 2012a,b) as well as the activity of STs (Papenbrock and Schmidt, 2000; Brychkova et al., 2013). Protein extracts were diluted at a ratio of 1:25 with Milli-Q water and mixed with diluted solutions of the Bio-Rad Protein Assay (Bio-Rad; www.bio-rad.com/), according to Bradford (1976), at a ratio of 1:10. Absorbance for each sample was measured at 595 nm in an Epoch Microplate Spectrophotometer with Gen 5 1.10 software (BioTek; www.biotek.com/).

### Sulfur Transferase Enzymes Activity Measurement

#### Sulfite Consuming Activity

Sulfite-consuming activity by the STs was determined as described before (Papenbrock and Schmidt, 2000; Tsakraklides et al., 2002) with modifications. The reaction assay contained 0.1 M Tris-HCl buffer, pH 9.5, 0.5 mM Na_2_SO_3_, 5 mM β-mercaptoethanol, 50 μM NaSCN, and 80 μg mL^−1^ of desalted protein extract-treated before the assay with 10 mM sodium orthovanadate (Kaufholdt et al., 2015) for 30 min at 4°C to disrupt SO activity that consumes sulfite. The sulfite-consuming activity was measured for 15 min at 30°C and was estimated as sulfite disappearance, as described above for SO activity and employing Na_2_SO_3_ as a standard solution containing NaSCN. Sulfite-consuming activity is expressed as nmol sulfite min^−1^ mg^−1^ protein.

#### Sulfide (H_2_S) Generation Activity

STs' sulfide-generation activity was determined as described before by Papenbrock and Schmidt (2000) with slight modifications. Assay mixtures of 200 μl contained 0.1 M Tris-HCl, pH 9.0, 2.5 mM dithiothreitol, and 10-μg protein extracts, and were started by adding 200 μM sodium thiosulfate. Reactions were incubated for 20 min at 37°C (T20), at the same time, proteins were incubated without 200 μM sodium thiosulfate as a blank (T0). The amount of H_2_S developed during the reaction was fixed by adding 20 μl 30 mM FeCl_3_ dissolved in 1.2 M HCl and 20 μl 20 mM NN dimethyl-p-phenylene-diamine dissolved in 7.2 M HCl. Samples were kept in the dark for 40 min, centrifuged, and the absorption of methylene blue formed was measured at 670 nm.

#### Sulfite Producing Activity

Sulfite generating STs activity was determined by colorimetric detection by measuring SCN formation as the red Fe(SCN)_3_ complex from cyanide and thiosulfate (Papenbrock and Schmidt, 2000). The reaction mixture contained 0.1 M Tris-HCl buffer, pH 9, 10 mM KCN, 5 mM β-mercaptoethanol, 50 μg mL^−1^ desalted protein extract, and was initiated by adding 5 mM Na_2_S_2_O_3_. After incubation at 37°C for 40 min, the reaction was stopped by adding 40 μL acidic iron reagent (FeCl3, 50 g L^−1^; 65% HNO3, 200 ml L^−1^) centrifuged at 8,000 g for 3 min and was read at 460 nm using an acidic iron reagent. Spontaneous rates of thiocyanate formation were determined by omitting the crude extract from the reaction mixture. Amounts of product formation were quantified using a standard curve done with NaSCN.

#### Quantitative Real-Time RT-PCR

Real-time PCR evaluated transcript analysis of specific genes in WT and SiR, SO modified single and double mutant plants. Total RNA was isolated using an Aurum™ Total RNA Mini Kit (BioRad; www.bio-rad.com/). The concentration and the purity of RNA were determined by spectrophotometry (NanoDrop 1000 Spectrophotometer). The integrity and size distribution of total RNA was checked by agarose gel electrophoresis with ethidium bromide staining. According to the manufacturer orders, reverse transcription was performed with 350ng of total RNA in 10 μl volume, using an iScriptTM cDNA synthesis kit (BioRad). PCR amplification was carried out with double-strand DNA-specific dye SYBR Green, using IQ (BioRad; www.bio-rad.com/). Amplification was monitored in real-time using an iCycler IQ multicolor real-time PCR Detection System (BioRad). The list of primers (Supplementary Table 1) was designed to overlap at least one exon junction. Ct (ΔCt) differences between the target transcript and ACTIN2 (At3g18780) as the housekeeping gene were calculated, and Ct values for the control and target samples were compared.

### Metabolite Analysis

A specific sulfite detection assay detected sulfite based on chicken SO as Brychkova et al. (2012a) described. Sulfate contents in plant material were quantified by ion chromatography (Dionex, ICS-5000). For sample processing, 20 mg of frozen plant material were extracted in 1 mL ultrapure Milli-Q water, centrifuged for 10 min at 21,000 g at 4°C twice, and then the supernatant was diluted 1:4 with Milli-Q water. To determine the total S content, 5 mg of dried and powdered plant material were placed into the tubes and digested in 70% nitric acid by heating at 170°C for 2 h. The amounts of total sulfur were quantified by inductively coupled plasma emission spectrometry (ICP-AES). Total non-protein thiol content was detected by using 5,5'-dithiobis (2-nitro-benzoic acid) (DTNB) as described by De Kok et al. (1997). The frozen plant material was extracted 1:10 in extraction buffer (8 mM ascorbic acid, 80 mM sulfosalicylic acid, and 1 mM EDTA). The extract was deproteinized by heating at 100°C for 4 min and centrifuging for 20 min at 21,000 g at 0°C. Water-soluble thiols in the supernatant were measured as a colored product of the reaction DTNB with sulfhydryl groups at 412 nm. Organic sulfur was calculated as the difference between total S to the inorganic S (sulfate + sulfite). Other sulfur compounds were calculated by subtracting sulfite, sulfate, and total glutathione from total sulfur. Glutathione was determined by the reusing assay described before by Tietze (1969). The method relies on the Glutathione Reductase (GR) dependent reduction of DTNB monitored at 412 nm and measures “total glutathione” = reduced glutathione (GSH) plus GSSG. Measurement of GSSG was achieved by pre-treatment of 200 μL of extract aliquots with 5 μL of 2-vinylpyridine (VPD) for 20 min (unless stated otherwise) at room temperature as described by Griffith (1980). GR was freshly prepared in 0.12 M NaH_2_PO_4_ (pH 7.5) and 6 mM EDTA. To measure total glutathione, quadruplicate aliquots of 20 μl (unless stated otherwise) neutralized extract was added to plate wells containing 0.175 ml of 0.12 M NaH_2_PO_4_ (pH 7.5), 6 mM EDTA, 5 μl of 20 mM NADPH, 20 μl of 6 mM DTNB. The addition of 5 μL GR started the reaction. The increase in 412 nm was monitored for 5 min by employing an Epoch Microplate Spectrophotometer with Gen5 1.10 software. Standards were run concurrently in the same plates. The same principle measured GSSG after incubation with VPD. To remove excess VPD, the derivatized solution was centrifuged thrice, and triplicate 20 μl aliquots (unless stated otherwise) of the final supernatant were assayed as described above. GSSG standards run concurrently were subjected to the same VPD derivatization as the extracts. The MDA level in plant tissue was measured as described by Srivastava et al. (2017). Anthocyanin level in plant tissue was measured as described by Laby et al. (2000).

### Statistical Analysis

The data represent means of three independent experiments or representative data of one of at least three independent experiments with similar results. Statistical analyses were performed by one- or two-way analysis of variance (ANOVA) according to the experimental layout. Subsequently, mean comparison of the different attributes was carried out by Tukey-Kramer honestly significant difference mean-separation test (Tukey-Kramer HSD test; JMP 8.0 software; SAS Institute Inc; www.jmp.com/), which was used to compare multiple groups of samples.

## Data Availability Statement

The original contributions presented in the study are included in the article/Supplementary Material, further inquiries can be directed to the corresponding author/s.

## Author's Note

The sulfite oxidase (SO) enzyme is an important player in protecting plants against exogenous applied and endogenously generated toxic sulfites. The role of SO in maintaining optimal carbon and sulfur metabolism was not yet fully established. By comparing *Arabidopsis* WT to Sulfite reductase and SO impaired plants grown with normal, excess, or limited sucrose or sulfate in the growth medium, we show the importance of active SO in carbon and sulfur metabolism. Notably, in the absence of active SO, the limited or excess sucrose or sulfate led to sulfite increase and enhanced oxidative stress that affected biomass accumulation in the mutant compared with WT plants. This is attributed to the channeling of the excess unoxidized sulfite toward the sulfate reduction pathway, resulting in futile reductant consumption and accumulation of excess organic sulfur. The results indicate that the role of SO in *Arabidopsis* plants is not limited to protection against elevated sulfite but to maintaining optimal carbon and sulfur metabolism under non-optimal sulfur or carbon availability.

## Author Contributions

DO participated in designing the research plans, performed the experiments, and analyses. AK participated in work to determine sulfite. AB cooperated with APR activity. AS participated in relative expression analysis. ZN cooperated with immunoblots analysis. AD participated in malondialdehyde (MDA) measurement. DS read and commented on the manuscript. MS conceived the original idea, designed the research plan, and supervised the research work. The manuscript was jointly written by DO and MS. All authors contributed to the article and approved the submitted version.

## Conflict of Interest

The authors declare that the research was conducted in the absence of any commercial or financial relationships that could be construed as a potential conflict of interest.

## Supplementary Material

Supplementary Figure 1 The effect of starvation (20 μM), normal (0.85 mM) and excess (4 mM) sulfate treatment on S containing metabolites, on expression levels of the sulfur dioxygenase ethylmalonic encephalopathy protein1 (ETHE1) in wild-type (WT) and (SiR) and sulfite oxidase (SO) impaired plants. **(A)** Non-protein soluble thiol contents **(B)**. Oxidized glutathione (GSSG). **(C)** Expression levels of ETHE1 transcript. The expression of each treated line was compared with the WT grown with 0.85 mM sulfate treatment after normalization to ACTIN2 gene product (At3g18780). Values represent one of three independent experiments with similar results (±SE, *n* = 3). Different lower-case letters indicate differences between genotypes within the same treatment. Different uppercase letters indicate significant differences within the plant genotypes in response to treatment (Tukey–Kramer HSD test; JMP 8.0 software).

Supplementary Figure 2 The effect of starvation (free) and normal (0.5%) sucrose treatment on total sulfur (S) in wild-type (WT) and SiR (SiR KD), SO (SO Ri) modified plants. Values represent the means of three independent experiments (±SE, *n* = 3). Different lowercase letters indicate differences between genotypes within the same treatment. Different uppercase letters indicate significant differences within the plant genotypes in response to treatment (Tukey–Kramer HSD test; JMP 8.0 software).

Supplementary Figure 3 Characterization of *Arabidopsis* wild-type (WT) and SiR (SiR KD), SO (SO Ri) modified plants in response to normal (0.5%), starved (sucrose free), and excess (3%) amount of carbon. **(A)** Response of WT and SiR, SO modified plants to carbon applications. Plants were photographed at 9 days of exposure. **(B)** Total biomass accumulation of the upper part of the plant in WT, SiR KD, and SO Ri plants grown under three different levels of carbon, FW. Values are means (*n* = 4 replications each with five plants). Different lowercase letters indicate differences between genotypes within the same treatment. Different uppercase letters indicate significant differences within the plant genotypes in response to treatment (Tukey–Kramer HSD test; JMP 8.0 software).

Supplementary Figure 4 Schematic illustration describing the impact of sulfite oxidase (SO) and sulfite reductase (SiR) impairment on the sulfur metabolism in *Arabidopsis* plants grown under excess (3%) sucrose conditions. Differently colored asterisks mean significant differences between SO Ri/SiR KD mutants and WT within a treatment. The presented significant differences are based on statistical analyses shown in Figure 4 and Table 1. The organic S was calculated as the difference between total S to the inorganic S (sulfate + sulfite). ATPS, adenosine phosphate sulfurylase; APS, adenosine-5′-phosphosulfate; APR, APS reductase; SiR, sulfite reductase; OAS-TL, O-acetyl-serine-thiol-lyase; SO, sulfite oxidase; STs, sulfur transferases; Cys, cysteine; Fdox, Oxidized ferredoxin; Fdred, reduced ferredoxin; H_2_O_2_, hydrogen peroxide; PPi, diphosphate; DTT, dithiothreitol; DTTox, Oxidized dithiothreitol.
