# Supplementary material for: Level of Sulfite Oxidase Activity Affects Sulfur and Carbon Metabolism in Arabidopsis
Source: Front Plant Sci. 2021 Jun 24;12:690830. doi: 10.3389/fpls.2021.690830 (PMC8264797; doi:10.3389/fpls.2021.690830)
Supplement: Supplementary file 1 [file Table_1.DOCX]

**Supplementary table S1**. List of gene-specific primers for gene expression analysis

| **Transcript** | **Accession number** | **Primer sequence (5’->3’)** | **PCR product, bp** |
| --- | --- | --- | --- |
| *ETHE1* | AT1G5358 | GACTTTCAGGAAGGAAGCTCAGATCA  TCTTTAGTTAGACGCGGGTTGTGTTG | 161 |
