# Supplementary figures and images for: Level of Sulfite Oxidase Activity Affects Sulfur and Carbon Metabolism in Arabidopsis
Source: Front Plant Sci. 2021 Jun 24;12:690830. doi: 10.3389/fpls.2021.690830 (PMC8264797; doi:10.3389/fpls.2021.690830)

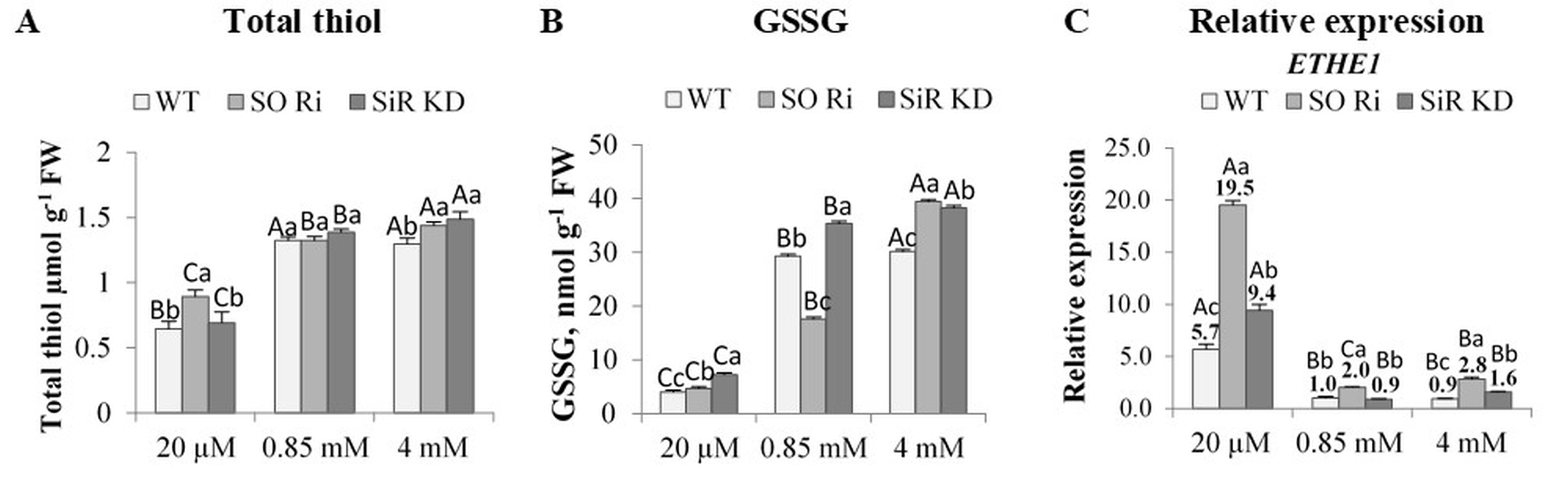

Supplement: Supplementary Figure 1 — The effect of starvation (20 μM), normal (0.85 mM) and excess (4 mM) sulfate treatment on S containing metabolites, on expression levels of the sulfur dioxygenase ethylmalonic encephalopathy protein1 (ETHE1) in wild-type (WT) and (SiR) and sulfite oxidase (SO) impaired plants. (A) Non-protein soluble thiol contents (B). Oxidized glutathione (GSSG). (C) Expression levels of ETHE1 transcript. The expression of each treated line was compared with the WT grown with 0.85 mM sulfate treatment after normalization to ACTIN2 gene product (At3g18780). Values represent one of three independent experiments with similar results (±SE, n = 3). Different lower-case letters indicate differences between genotypes within the same treatment. Different uppercase letters indicate significant differences within the plant genotypes in response to treatment (Tukey–Kramer HSD test; JMP 8.0 software). [file Image_1.TIF]

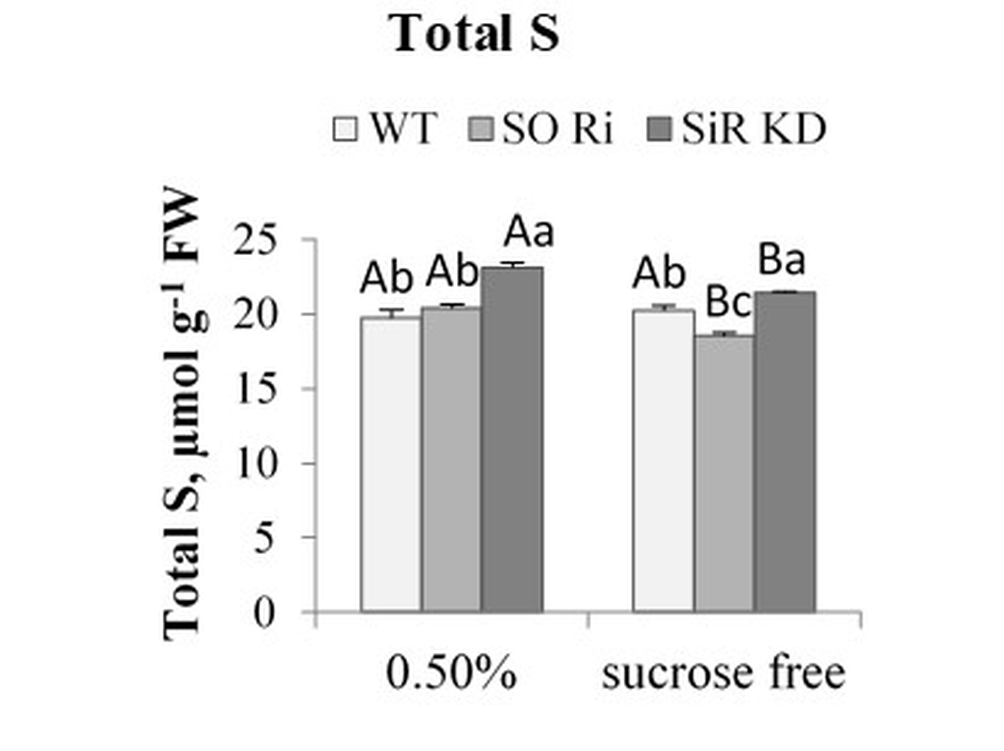

Supplement: Supplementary Figure 2 — The effect of starvation (free) and normal (0.5%) sucrose treatment on total sulfur (S) in wild-type (WT) and SiR (SiR KD), SO (SO Ri) modified plants. Values represent the means of three independent experiments (±SE, n = 3). Different lowercase letters indicate differences between genotypes within the same treatment. Different uppercase letters indicate significant differences within the plant genotypes in response to treatment (Tukey–Kramer HSD test; JMP 8.0 software). [file Image_2.TIF]

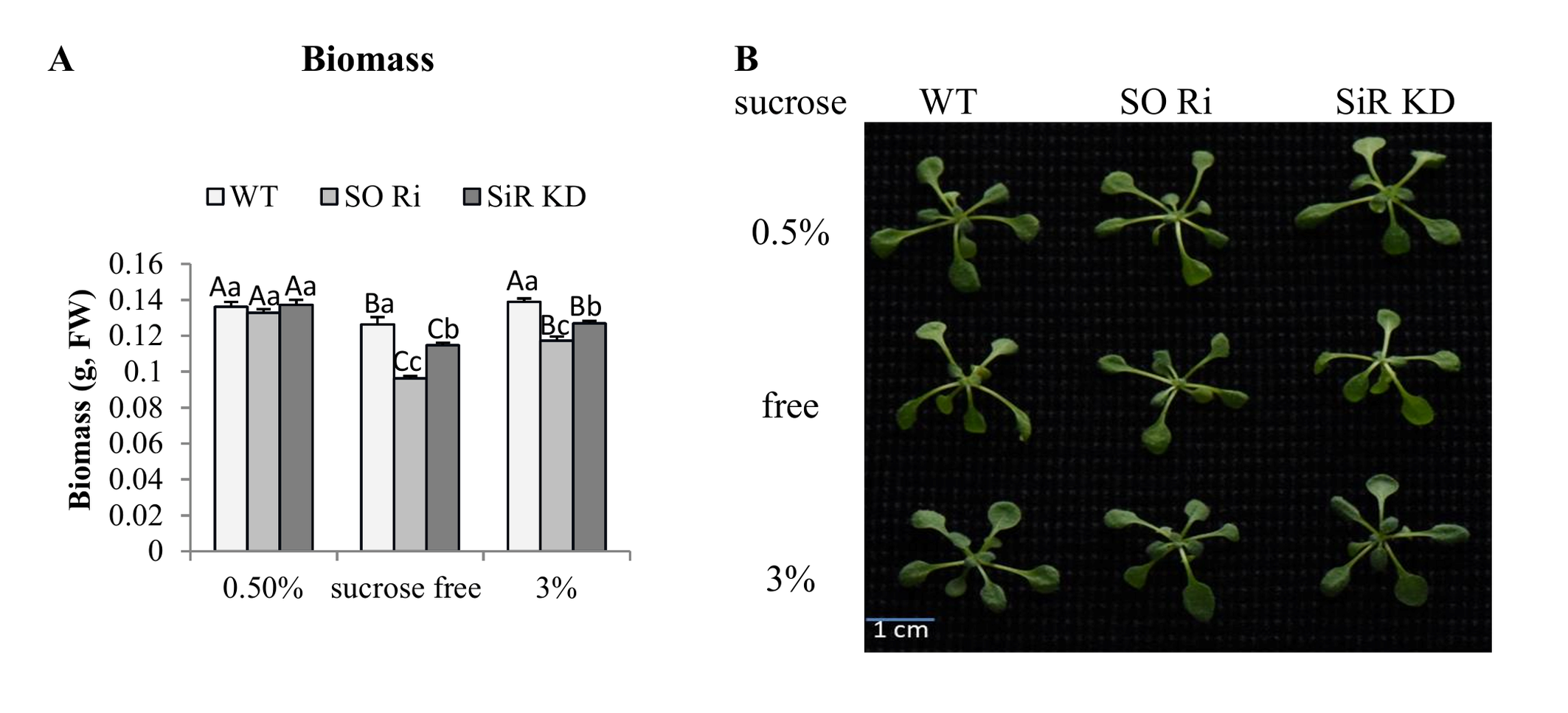

Supplement: Supplementary Figure 3 — Characterization of Arabidopsis wild-type (WT) and SiR (SiR KD), SO (SO Ri) modified plants in response to normal (0.5%), starved (sucrose free), and excess (3%) amount of carbon. (A) Response of WT and SiR, SO modified plants to carbon applications. Plants were photographed at 9 days of exposure. (B) Total biomass accumulation of the upper part of the plant in WT, SiR KD, and SO Ri plants grown under three different levels of carbon, FW. Values are means (n = 4 replications each with five plants). Different lowercase letters indicate differences between genotypes within the same treatment. Different uppercase letters indicate significant differences within the plant genotypes in response to treatment (Tukey–Kramer HSD test; JMP 8.0 software). [file Image_3.TIF]

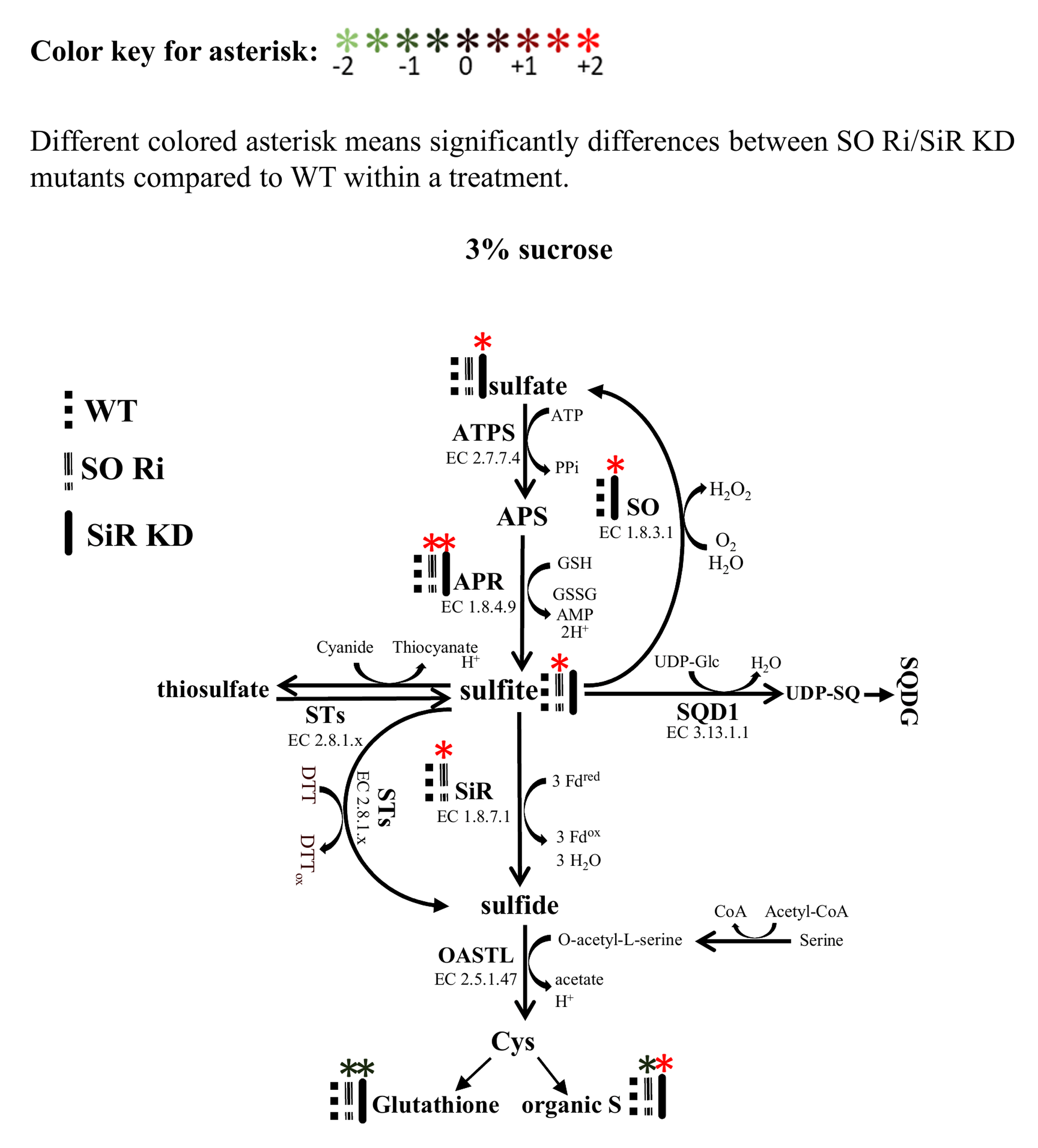

Supplement: Supplementary Figure 4 — Schematic illustration describing the impact of sulfite oxidase (SO) and sulfite reductase (SiR) impairment on the sulfur metabolism in Arabidopsis plants grown under excess (3%) sucrose conditions. Differently colored asterisks mean significant differences between SO Ri/SiR KD mutants and WT within a treatment. The presented significant differences are based on statistical analyses shown in Figure 4 and Table 1. The organic S was calculated as the difference between total S to the inorganic S (sulfate + sulfite). ATPS, adenosine phosphate sulfurylase; APS, adenosine-5′-phosphosulfate; APR, APS reductase; SiR, sulfite reductase; OAS-TL, O-acetyl-serine-thiol-lyase; SO, sulfite oxidase; STs, sulfur transferases; Cys, cysteine; Fdox, Oxidized ferredoxin; Fdred, reduced ferredoxin; H2O2, hydrogen peroxide; PPi, diphosphate; DTT, dithiothreitol; DTTox, Oxidized dithiothreitol. [file Image_4.TIF]
